# Stroke Prevention in Atrial Fibrillation: A Clinical Perspective on Trials of the Novel Oral Anticoagulants

**DOI:** 10.1007/s10557-015-6632-3

**Published:** 2016-01-18

**Authors:** João Morais, Raffaele De Caterina

**Affiliations:** Cardiology Division, Santo Andre’s Hospital, Pousos, 2410-197 Leiria Portugal; Cardiology, Ospedale SS. Annunziata, G. d’Annunzio University, Chieti, Italy

**Keywords:** Anticoagulation, Apixaban, Atrial fibrillation, Dabigatran, Rivaroxaban, Stroke prevention

## Abstract

Atrial fibrillation (AF) is a common heart rhythm disturbance; its incidence increases with age, and it is also an independent risk factor for stroke. Anticoagulation has been proven as the most effective way to reduce the risk of stroke in patients with AF, and vitamin K antagonists have been used for decades as the gold standard treatment. Vitamin K antagonists have a narrow therapeutic window in addition to variable pharmacokinetics and pharmacodynamics, and they frequently interact with food and other drugs, requiring coagulation monitoring to ensure balance between safety and efficacy. The novel oral anticoagulants (NOACs) dabigatran, rivaroxaban, apixaban, and edoxaban selectively target either thrombin or Factor Xa and have predictable pharmacologic profiles, removing the need for routine coagulation monitoring. This article summarizes phase III data in patient subtypes and discusses controversies surrounding AF management with these agents. Results indicate that NOACs in non-valvular AF have an overall improved efficacy–safety profile compared with warfarin. Significantly fewer fatal bleeding events were observed in patients randomized to rivaroxaban, apixaban, or edoxaban compared with those on warfarin, and significant reductions in the incidence of life-threatening bleeding were observed in patients randomized to dabigatran. All four pivotal trials testing the NOACs against warfarin showed significantly lower rates of intracranial bleeding in patients administered NOACs. These results suggest that wider use of NOACs has the potential to improve outcomes for most patients with AF.

## Introduction

Atrial fibrillation (AF) is a common cardiac arrhythmia that increases in prevalence with advancing age and confers a fivefold increase in the risk of stroke. Blood stasis (usually in the left atrium) caused by AF may lead to the formation of a clot, which can then embolize. Approximately every fifth stroke is attributable to AF, and the risk of stroke is age related. In addition, AF-related strokes are frequently severe and result in disability and mortality more often than non-AF-related strokes [1]. The elevated risk of stroke in patients with AF is generally managed with oral anticoagulants (OACs), such as vitamin K antagonists (VKAs). Although highly effective, VKAs have limitations (Table 1), and novel oral anticoagulants (NOACs, also known as direct OACs or non-VKA OACs) such as dabigatran, rivaroxaban, apixaban, and edoxaban were developed with the aim of overcoming some of these limitations (Table 2). These agents have demonstrated equivalent or superior efficacy and better safety profiles compared with VKAs in stroke prevention phase III trials and are increasingly being integrated into routine clinical practice. This review will outline the key efficacy, safety, and tolerability results from the phase III stroke prevention studies of NOACs. The article will also evaluate the clinical management of patients in high-risk subgroups and issues that may be faced by clinicians prescribing anticoagulants.

**Table 1:** Novel oral anticoagulants: summary of advantages and disadvantages compared with warfarin

Advantages	✓ Fixed dosing ✓ Lack of clinically meaningful dietary interactions ✓ Predictable pharmacology ✓ Far fewer drug interactions ✓ No need for routine coagulation monitoring or regular dose adjustment ✓ Wider therapeutic range ✓ Fast on–off action eliminates need for bridging therapy with parenteral anticoagulants ✓ Shorter half-lives beneficial in urgent surgery situations ✓ Reduction in rate of stroke and systemic embolism equal to or superior to warfarin in clinical trials ✓ Reduced rates of intracranial hemorrhage and fatal bleeding events in clinical trials
Disadvantages	✗ Tests for measuring NOAC concentrations are not widely available ✗ No specific antidotes available (vitamin K for warfarin is slow-acting) ✗ Contraindicated or not recommended in patients with severe renal failure and in patients with prosthetic heart valves ✗ In theory, the shorter half-lives of the NOACs might be a potential concern in non-compliant patients; however, there is a paucity of pertinent data ✗ Possible interactions with rate-/rhythm-controlling drugs such as amiodarone and verapamil ✗ Scarce data about concomitant use of dual antiplatelet therapy, or use in patients with both atrial fibrillation and acute coronary syndrome

*NOAC* novel oral anticoagulant

**Table 2:** Novel oral anticoagulants either approved or in development for stroke prevention in patients with atrial fibrillation

	Direct thrombin inhibitor	Direct factor Xa inhibitors		
	Dabigatran [2, 3]	Rivaroxaban [4–7]	Apixaban [8–10]	Edoxaban [11–13]
Prodrug	Dabigatran etexilate	No	No	No
Oral bioavailability	~6–7 %	80–100 %^a^	50 %	~61 %
Plasma protein binding	35 %	92–95 %	87 %	40–59 %
Half-life (h)	12–14	5–9 (young) 11–13 (elderly)	8–13.5	6–11
Time to maximum plasma concentration (h)	1.25–3	2–4	3–4	1–2
Renal clearance^b^	85 %^c^	33 %	27 %	35–39 %

^a^15 mg and 20 mg doses to be taken with food

^b^Of active unchanged drug as a proportion of administered dose

^c^After intravenous administration

## The Role of Oral Anticoagulants in Preventing Stroke in Patients with Atrial Fibrillation

VKAs such as warfarin, phenprocoumon, and acenocoumarol reduce the rate of AF-related stroke by approximately 40 % relative to acetylsalicylic acid (ASA) [14]. However, owing to the narrow therapeutic range, large intra-individual variation in response, unpredictable pharmacology, and numerous drug interactions associated with VKA use, routine coagulation (international normalized ratio) monitoring is required to avoid an excess risk of cerebrovascular events [15]. Together with diet and lifestyle restrictions [15], this negatively impacts on patients’ quality of life, contributes to under-prescribing, and is responsible for poor compliance in some patients [16]. Real-world evidence from patients with newly diagnosed non-valvular AF enrolled in cohort one of GARFIELD-AF (between 2009 and 2011, before widespread approval of the NOACs) indicates that under-prescribing of OACs and over-reliance on antiplatelet agents is commonplace; approximately 40 % of patients did not receive OACs (against guideline recommendations), and the majority of these patients (around two-thirds) received antiplatelet agents alone [17].

The choice of any antithrombotic agent is based on achieving a balance between the patient’s risks of thromboembolism and bleeding. Current international guidelines in AF advocate use of the CHADS_2_ (1 point each for Congestive heart failure, Hypertension, Age ≥75 years, and Diabetes mellitus, and 2 points for prior Stroke/transient ischemic attack) [18] and the CHA_2_DS_2_-VASc (as CHADS_2_, but 2 points for Age ≥75 years and 1 point each for Vascular disease, Age 65–74 years, and Sex category female) scores for assessing stroke risk [19, 20]. In the European Society of Cardiology (ESC) 2012 guidelines update, the HAS-BLED (Hypertension, Abnormal renal/liver function, Stroke, Bleeding history or predisposition, Labile international normalized ratio, Elderly [age >65 years], Drugs/alcohol concomitantly) score is also recommended to identify patients at a high risk of bleeding (score ≥3) [20]. In general, oral anticoagulation is recommended for all patients with AF, except for low-risk patients with a CHADS_2_ or CHA_2_DS_2_-VASc score of 0 (including women aged <65 years with no other risk factors), who do not need any antithrombotic therapy [20]. Although older guidelines (including the American College of Chest Physicians [ACCP] 2012 guidelines) included recommendations for ASA as an antithrombotic agent suitable for stroke prevention in high-risk patients [18], the role of ASA (and other antiplatelet agents) is diminished in the latest major European and American guidelines. The updated ESC 2012 guidelines no longer recommend the use of antiplatelet agents for stroke prophylaxis (except in patients refusing OAC therapy), and the 2014 American Heart Association (AHA) guidelines reserve it as an alternative to oral anticoagulation or to no therapy in patients with a CHA_2_DS_2_-VASc score of 1 [19, 20]. The balance between anticipated benefit and potential risk of bleeding with OAC therapy should be considered on an individual basis. However, according to the guidelines, bleeding risk alone should not be used to exclude patients for anticoagulation, but should draw attention to modifiable risk factors affecting the risk of bleeding.

## How Effective Are Novel Oral Anticoagulants for Stroke Prevention in Atrial Fibrillation? Results from Phase III Trials

Large phase III trials for stroke prevention in patients with non-valvular AF have been completed for dabigatran (RE-LY [21]), rivaroxaban (ROCKET AF [22]), apixaban (ARISTOTLE [23] and AVERROES [24]), and edoxaban (ENGAGE AF-TIMI 48 [25]) (Table 3). Results from all trials point to an efficacy similar or superior to warfarin or ASA (Table 4).

**Table 3:** Key features of phase III trials with novel oral anticoagulants

	RE-LY [21, 26] (dabigatran)	ROCKET AF [22] (rivaroxaban)	ARISTOTLE [23] (apixaban)	ENGAGE AF [25] (edoxaban)
Patients randomized	18,113	14,264	18,201	21,105
Study blinding	Double-blind, randomized dose of dabigatran; open-label warfarin	Double-blind, double-dummy	Double-blind, double-dummy	Double-blind, double-dummy, randomized dose of edoxaban
Study design	Non-inferiority, prespecified hierarchical superiority testing			
Comparator	Dose-adjusted warfarin (target INR 2–3)			
Doses tested	110 mg bid or 150 mg bid	20 mg od (15 mg od for patients with CrCl 30–49 mL/min)	5 mg bid (2.5 mg bid for patients with ≥2 of: weight ≤60 kg, age ≥80 years, or serum creatinine ≥1.5 mg/dL)	60 mg od or 30 mg od (dosage halved for patients with CrCl 30–50 mL/min, body weight ≤60 kg, or concomitant use of verapamil, quinidine, or dronedarone)

*bid* twice daily, *CrCl* creatinine clearance, *INR* international normalized ratio, *od* once daily

**Table 4:** Key efficacy results from phase III trials (intention to treat) with novel oral anticoagulants compared with standard therapy (rates per 100 patient-years)

	RE-LY [21, 26, 27] (dabigatran)^a^		ROCKET AF [22] (rivaroxaban)	ARISTOTLE [23] (apixaban)	ENGAGE AF [25] (edoxaban)	
	110 mg bid	150 mg bid			30 mg od	60 mg od
Stroke or SE (%/year)	1.54 vs 1.72^†^	1.12 vs 1.72***	2.1 vs 2.4^†^	1.27 vs 1.60**	2.04 vs 1.80^†^	1.57 vs 1.80^†^
Ischemic stroke (%/year)	1.34 vs 1.22^†,b^	0.93 vs 1.22*^,b^	1.34 vs 1.42^†^	0.97 vs 1.05^†,b^	1.77 vs 1.25***	1.25 vs 1.25^†^
Hemorrhagic stroke (%/year)	0.12 vs 0.38***	0.10 vs 0.38***	0.26 vs 0.44*	0.24 vs 0.47***	0.16 vs 0.47***	0.26 vs 0.47***

*bid* twice daily, *od* once daily, *SE* systemic embolism

^†^ *p* = not significant; * *p* < 0.05; ** *p* ≤ 0.01; *** *p* < 0.001

^a^ Updated data (2010 and 2014) after identification of additional events post-publication (2009)

^b^ Ischemic or uncertain type of stroke

Dabigatran, rivaroxaban, apixaban, and edoxaban all demonstrated non-inferiority to warfarin with respect to the primary efficacy endpoint, the composite of stroke and systemic embolism. Dabigatran 150 mg twice daily (bid) and apixaban 5 mg bid also demonstrated superiority to warfarin for the primary efficacy endpoint in the intention-to-treat (ITT) population (hazard ratios [HRs], 0.65 and 0.79, respectively) [21, 23, 27]. Rivaroxaban 20 mg once daily (od) was superior to warfarin while patients were receiving treatment (HR, 0.79; *p* = 0.02) and was non-inferior in the ITT analysis, which included events occurring after early discontinuation of the study drugs [22]. For edoxaban, a modified ITT analysis, including all patients receiving at least one dose of the drug, showed that both the 30 mg and 60 mg od regimens of edoxaban were non-inferior for the primary efficacy endpoint compared with well-managed warfarin (median time in therapeutic range = 68.4 % of the treatment period) (*p* for non-inferiority = 0.005 and *p* < 0.001, respectively) [25]. Overall, for the prevention of ischemic stroke, only dabigatran 150 mg bid was superior to warfarin [21]. All agents significantly reduced rates of hemorrhagic stroke relative to warfarin.

AVERROES, the superiority phase III trial of apixaban versus ASA in patients considered ‘unsuitable’ for VKAs, demonstrated that apixaban is an effective alternative to ASA. AVERROES was stopped after 1.1 years of follow-up because of the clear superiority of apixaban over ASA for the primary endpoint, with similar rates of major bleeding (including intracranial hemorrhage [ICH]) [24]. The benefit–risk profile of apixaban versus ASA, as demonstrated in the AVERROES trial, reinforces the latest guideline recommendations that ASA should no longer be considered a suitable alternative to OACs for stroke prevention in the majority of patients with non-valvular AF.

One in four patients who experience an AF-related stroke die within 30 days of the index event [28]. VKA treatment reduces overall mortality by 26 % relative to placebo [14], and it is notable that all NOACs tested in phase III trials also demonstrated a strong trend towards reduced all-cause mortality in the ITT population compared with warfarin; this was statistically significant only for apixaban versus warfarin (HR, 0.89; *p* = 0.047) [23] and was close to statistical significance for the 150 mg bid dose of dabigatran (relative risk, 0.88; *p* = 0.051) [21].

A meta-analysis by Ruff et al. [29] of all 71,683 participants included in the RE-LY, ROCKET AF, ARISTOTLE, and ENGAGE AF-TIMI 48 trials found that allocation to a NOAC significantly reduced the composite of stroke or systemic embolism by 19 % compared with patients receiving warfarin. This overall reduction was largely driven by the 51 % reduction in the incidence of hemorrhagic stroke among patients treated with a NOAC. Compared with warfarin, NOACs were also associated with a significant 10 % reduction in all-cause mortality [29].

## Bleeding Risk on Anticoagulation: Safety and Tolerability Profiles of Novel Oral Anticoagulants in Phase III Trials

Balancing stroke prevention against the risk of major or severe bleeding is complicated by the fact that several stroke risk factors (such as hypertension, prior stroke, and chronic renal dysfunction) are also bleeding risk factors [20]. VKA treatment increases the risk of ICH approximately twofold compared with ASA [14], but a key finding from all of the phase III trials was that the incidence of major bleeding events with NOAC treatment was similar to or lower than with warfarin [21–23, 25].

The principal safety outcome was major bleeding (RE-LY, ARISTOTLE, AVERROES, ENGAGE AF) or the composite of major bleeding and non-major clinically relevant bleeding (ROCKET AF; Table 5). In RE-LY, dabigatran 150 mg bid demonstrated similar rates of major bleeding compared with warfarin, whereas dabigatran 110 mg bid demonstrated improved safety outcomes compared with warfarin, reducing rates of major bleeding by 20 % [21]. In ROCKET AF, the rates of major and non-major clinically relevant bleeding were similar in patients receiving rivaroxaban compared with those given warfarin [22]. Apixaban demonstrated superiority in terms of primary safety outcomes compared with warfarin in the ARISTOTLE trial, reducing the rate of major bleeding by 31 % [23]. In AVERROES, patients receiving apixaban had rates of major bleeding similar to patients receiving ASA (1.4 %/year vs 1.2 %/year) [24]. Edoxaban 30 mg od and 60 mg od doses were both associated with lower rates of major bleeding (*p* < 0.001 for both doses) and life-threatening bleeding (*p* < 0.001 for both doses) compared with warfarin in the ENGAGE AF trial [25].

**Table 5:** Key safety results from phase III trials with novel oral anticoagulants compared with standard therapy

	RE-LY [21, 26, 27] (dabigatran)^a^		ROCKET AF [22, 30] (rivaroxaban)	ARISTOTLE [23] (apixaban)	ENGAGE AF [25] (edoxaban)	
	110 mg bid	150 mg bid			30 mg od	60 mg od
Major bleeding (%/year)	2.92 vs 3.61**	3.40 vs 3.61^†^	3.60 vs 3.40^†^	2.13 vs 3.09***	1.61 vs 3.43***	2.75 vs 3.43***
Major and NMCR bleeding (%/year)	N/A	N/A	14.90 vs 14.50^†^	4.07 vs 6.01***	7.97 vs 13.02***	11.10 vs 13.02***
Major GI bleeding (%/year)	1.15 vs 1.07^†^	1.56 vs 1.07***	2.00 vs 1.24***	0.76 vs 0.86^†^	0.82 vs 1.23***	1.51 vs 1.23*
Intracranial hemorrhage (%/year)	0.23 vs 0.76***	0.32 vs 0.76***	0.50 vs 0.70*	0.33 vs 0.80***	0.26 vs 0.85***	0.39 vs 0.85***
All-cause mortality (%/year)	3.75 vs 4.13^†^	3.64 vs 4.13^†^	4.50 vs 4.90^†^	3.52 vs 3.94*	3.80 vs 4.35**	3.99 vs 4.35^†^
Myocardial infarction (%/year)	0.82 vs 0.64^†^	0.81 vs 0.64^†^	0.91 vs 1.12^†^	0.53 vs 0.61^†^	0.89 vs 0.75^†^	0.70 vs 0.75^†^

*bid* twice daily, *GI* gastrointestinal, *N/A* not applicable, *NMCR* non-major clinically relevant, *od* once daily

^†^ *p* = not significant; * *p* < 0.05; ** *p* < 0.01; *** *p* ≤ 0.001

^a^Updated data (2010 and 2014) after identification of additional events post-publication (2009)

Not all patients with AF should be treated with NOACs for stroke prevention. Dabigatran, rivaroxaban, apixaban, and edoxaban are contraindicated in patients with active pathologic bleeding or with a lesion or condition considered to be a significant risk of major bleeding, e.g., gastrointestinal (GI) ulceration. According to each NOAC’s European Summary of Product Characteristics, use is also contraindicated or not recommended in patients with end-stage renal disease [4, 8, 11, 31]. (Exceptionally, in the US, apixaban may be used in patients with end-stage renal disease [32].)

GI bleeding accounts for approximately 90 % of major bleeding events in patients with AF receiving VKAs [33]. Dabigatran 150 mg bid and rivaroxaban significantly increased rates of GI bleeding (1.5-fold) compared with warfarin in RE-LY and ROCKET AF, respectively [21, 22]. Apixaban was associated with GI bleeding rates that were similar to those with warfarin in ARISTOTLE (*p* = 0.37) [23]. GI bleeding also occurred more frequently with edoxaban 60 mg od than with warfarin in ENGAGE AF (*p* = 0.03), although edoxaban 30 mg od demonstrated significantly lower rates of GI bleeding compared with warfarin (*p* < 0.001) [25]. A pooled analysis of phase III trials of the NOACs found that, compared with warfarin, NOACs were associated with a 25 % increase in the incidence of GI bleeding (*p* = 0.04) [29].

The most devastating major bleeding complication associated with VKA treatment is ICH; the annualized hospitalization rate for warfarin-associated ICH is approximately 0.5 % [33]. Furthermore, the majority of warfarin-associated deaths are from ICH, and most ICH survivors have severe functional disability at discharge [33]. Patients taking NOACs have a lower risk of ICH compared with those prescribed warfarin. In phase III studies, dabigatran, rivaroxaban, apixaban, and edoxaban all significantly reduced the rate of ICH (by 33–70 %) compared with warfarin. It is probable that the decrease in ICH contributed to reductions in fatal/life-threatening bleeding and to the overall trend towards reduced mortality (Table 5). A meta-analysis demonstrated that, overall, NOACs reduced ICH by 52 % compared with warfarin (*p* < 0.0001) [29].

The positive trial data coupled with the increased convenience of fixed dosing have prompted AF guidelines to recommend the approved agents dabigatran, rivaroxaban, and apixaban as alternatives to warfarin, with a preference for the NOACs indicated in the European and Canadian guidelines [18–20, 34].

## Implications for Clinical Management of Specific Patient Populations

Although all patients with AF are at an elevated risk of stroke, some groups are considered more difficult to treat than others. The typical patient with AF is elderly with multiple co-morbidities [35]. These patients may be at higher risk of bleeding events than other groups [1] and may, therefore, be less likely to receive VKA treatment, even if their stroke risk is also higher [36]. The NOACs are effective alternatives to VKAs, and rivaroxaban, dabigatran, and apixaban are recommended by the ESC 2012 guidelines for the prevention of thromboembolism in patients with non-valvular AF and a CHA_2_DS_2_-VASc score of ≥1 [20]. These NOACs may hold promise in high-risk and challenging-to-treat patients; dose adjustments in specific patient groups according to approved labels for rivaroxaban, dabigatran, apixaban, and edoxaban are summarized in Table 6.

**Table 6:** Dosing in patients with atrial fibrillation for the approved novel oral anticoagulants dabigatran, rivaroxaban, apixaban, and edoxaban

	Dabigatran [31, 37]	Rivaroxaban [4, 38]	Apixaban [8, 32]	Edoxaban [11, 39]
Standard dose	EU, US: 150 mg bid (CrCl ≥30 mL/min)	EU, US: 20 mg od To be taken with food	EU, US: 5 mg bid	EU: 60 mg od US: 60 mg od – do NOT use in patients with CrCl >95 mL/min
Elderly				
Dose adjustments	EU: Age ≥80 years: 110 mg bid; in patients aged 75–80 years a reduction to 110 mg bid may be considered	–	EU, US: 2.5 mg bid in patients with ≥2 of the following criteria: age ≥80 years, body weight ≤60 kg, CrCl 15–50 mL/min	–
Renal impairment				
Dose adjustments	EU: 150 mg bid (reduction to 110 mg bid may be considered) (CrCl 30–49 mL/min) US: 75 mg bid (CrCl 15–30 mL/min or CrCl 30–50 mL/min and receiving concomitant treatment with dronedarone or systemic ketoconazole)	EU, US: 15 mg od (US: CrCl 15–50 mL/min; EU: CrCl 15–49 mL/min)	EU, US: 2.5 mg bid in patients with ≥2 of the following criteria: age ≥80 years, body weight ≤60 kg, serum creatinine ≥1.5 mg/dL (133 μmol/L) EU: 2.5 mg bid in patients with CrCl 15–29 mL/min US: No dose adjustment is recommended for patients with renal impairment alone, including those with end-stage renal disease maintained on hemodialysis	EU, US: 30 mg od (CrCl 15–50 mL/min)
Caution/avoid	–	EU: Use with caution in patients with CrCl 15–29 mL/min; not recommended in patients with CrCl <15 mL/min US: Avoid use in patients with CrCl <15 mL/min	EU: Use with caution in patients with CrCl 15–29 mL/min; not recommended in patients with CrCl <15 mL/min	EU, US: Not recommended in patients with CrCl <15 mL/min
Contraindications	EU: CrCl <30 mL/min US: No recommendation can be made for patients with CrCl <15 mL/min	–	–	–
Gastritis, esophagitis, gastroesophageal reflux				
Dose adjustments	EU: Consider dose reduction to 110 mg bid	–	–	–
Hepatic function				
Contraindications	EU: Hepatic impairment or liver disease expected to have an impact on survival	EU: Hepatic disease associated with coagulopathy and clinically relevant bleeding risk, including cirrhotic patients with Child–Pugh B and C, severe liver disease	EU: Hepatic disease associated with coagulopathy and clinically relevant bleeding risk, severe hepatic impairment	EU: Hepatic disease associated with coagulopathy and clinically relevant bleeding risk
Caution/avoid	EU: Not recommended in patients with elevated liver enzymes (>2 × ULN)	US: Avoid use in patients with moderate (Child–Pugh B) and severe (Child–Pugh C) hepatic impairment or with any hepatic disease associated with coagulopathy	EU: Mild or moderate hepatic impairment (Child–Pugh A or B), elevated liver enzymes ALT/AST >2 × ULN or total bilirubin ≥1.5 × ULN US: Not recommended in patients with severe hepatic impairment; no dosing recommendations provided for patients with moderate hepatic impairment	EU: Not recommended in patients with severe hepatic impairment; use with caution in patients with mild-to-moderate hepatic impairment US: Not recommended in patients with moderate-to-severe hepatic impairment

*ALT* alanine aminotransferase test, *AST* aspartate aminotransferase test, *bid* twice daily, *CrCl* creatinine clearance, *EU* European Union, *od* once daily, *ULN* upper limit of normal, *US* United States

### Elderly Patients

Patients with AF who are elderly are at a higher risk of both thromboembolic and bleeding events during anticoagulation treatment [1], but when the risks of anticoagulation are weighed against the advantages, these patients gain the greatest net clinical benefit from treatment [40]. Guidelines recommend anticoagulants over antiplatelet agents for elderly patients (≥75 years) [1, 18] because the thromboembolic efficacy of antiplatelet agents decreases with age [1].

In the phase III studies of NOACs, 31–44 % of enrolled patients were aged ≥75 years. As expected, rates of ischemic and hemorrhagic events were numerically higher in older patients than in younger patients, regardless of the treatment arm. In general, the benefits of NOACs in elderly patients were consistent with those observed in the overall study populations. In RE-LY, patients experienced similar rates of stroke/systemic embolism and ICH, regardless of age category [41]. There was a significant interaction between age and treatment (*p* ≤ 0.001) for major bleeding with both dabigatran doses, although this was observed only for extracranial bleeding. Younger patients (<75 years) experienced fewer major bleeding events with dabigatran relative to warfarin, whereas elderly patients (≥75 years) experienced similar or increased rates of bleeding with dabigatran relative to warfarin. Owing to this increased risk of bleeding in the elderly population, the European Union Summary of Product Characteristics for dabigatran etexilate recommends a dose reduction to 110 mg bid in patients ≥80 years [31]. In ROCKET AF, no significant interaction between age and treatment effect was observed for the primary efficacy endpoint, major bleeding, mortality, or ICH [42]. A small but significant interaction between age and treatment effect was, however, observed for clinically relevant non-major bleeding (rivaroxaban vs warfarin; patients aged ≥75 years, HR, 1.15; patients <75 years, HR, 0.94; interaction *p* = 0.01) [42]. Nevertheless, no dose adjustment for age is recommended in patients receiving this drug [4]. In ARISTOTLE, no significant interaction between age and treatment effect was observed for the primary efficacy endpoint (stroke or systemic embolism) or principal safety outcome (major bleeding) [23]. Prespecified outcomes in ARISTOTLE were investigated in relation to age in a separate analysis, demonstrating that apixaban was effective and well tolerated across all age groups (<65 years, 65 to <75 years, and ≥75 years), including patients ≥80 years (13 %) [43]. As per the study design for ARISTOTLE, the Summary of Product Characteristics for apixaban recommends a dose reduction to 2.5 mg bid in patients with at least two of the following risk factors: age ≥80 years, body weight ≤60 kg, or serum creatinine ≥1.5 mg/dL [8]. In the ENGAGE AF trial, the efficacy and safety of both doses of edoxaban compared with warfarin were consistent across age groups (<65 years, 65 to <75 years, and ≥75 years) [44]; consequently, no dose adjustment of edoxaban is required on the basis of age alone [11].

In summary, elderly patients may derive similar or even greater benefits from NOACs compared with the general population.

### Renal Impairment

Chronic renal disease is present in 10–15 % of patients with AF and may increase the risk of AF-related cardiovascular complications [1]. Clinical guidelines recommend baseline and subsequent regular assessments of renal function in patients after initiation of NOACs [19, 20]. Phase III trials of NOACs included 17–21 % of patients with moderate renal impairment (creatinine clearance [CrCl] 30–49 mL/min), but excluded patients with severe renal impairment (CrCl <30 mL/min for RE-LY [21], ROCKET AF [22], and ENGAGE AF [25]; CrCl <25 mL/min for ARISTOTLE and AVERROES [23, 24]). There was no dose adjustment in RE-LY, and patients were randomized to either the dabigatran 110 mg or 150 mg bid doses [21]. ROCKET AF prespecified a reduced dose for patients with moderate renal impairment (rivaroxaban 15 mg od), whereas in ARISTOTLE, patients with renal impairment (serum creatinine ≥1.5 mg/dL) received a reduced dose (apixaban 2.5 mg bid) only when ≥1 additional factors were present (age ≥80 years or body weight ≤60 kg) [23]. In ENGAGE AF, patients were randomized to either edoxaban 30 mg od or 60 mg od, and the edoxaban dose was subsequently halved in patients with an estimated CrCl of 30–50 mL/min at randomization or at any time during the study [25]. Patients with moderate renal impairment experienced numerically higher rates of ischemic and hemorrhagic events than patients with normal renal function, regardless of treatment. In the RE-LY, ROCKET AF, and ARISTOTLE trials no significant interactions between renal function and treatment effect were observed for stroke/systemic embolism prevention [45–47]. However, in ENGAGE AF, patients with CrCl >95 mL/min receiving edoxaban 60 mg od experienced twofold higher rates of ischemic stroke than those receiving warfarin; consequently, the US Prescribing Information states that edoxaban should not be used in patients with CrCl >95 mL/min and, according to the European Summary of Product Characteristics, edoxaban should only be used in patients with a high CrCl after careful evaluation of thromboembolic and bleeding risks [11, 39].

In RE-LY, no statistically significant interaction between treatment and renal function (calculated using the Cockcroft–Gault Formula) was observed for major bleeding; however, when renal function was calculated using either the Chronic Kidney Disease Epidemiology Collaboration (CKD-EPI) or the Modification of Diet in Renal Disease (MDRD) equation, significant interactions were observed: patients with high renal function (glomerular filtration rate ≥80 mL/min) experienced a greater relative reduction in major bleeding with either dabigatran dose compared with warfarin [45]. In ROCKET AF, no significant effects of renal function were observed for the principal safety outcome (interaction *p* = 0.45) or major bleeding (interaction *p* = 0.48). Fatal bleeding rates were also significantly lower in patients receiving rivaroxaban versus warfarin irrespective of renal function [46]. In ARISTOTLE, there was a greater reduction in major bleeding with apixaban compared with warfarin among patients with moderate or severe renal impairment (CrCl 25–49 mL/min) versus mild (CrCl 50–79 mL/min) or no renal impairment (*p* = 0.03 for interaction) [47].

Among the NOACs, renal excretion of the active unchanged drug ranges from 27 % to 85 % (Table 2). Because of decreased clearance and elevated plasma levels in patients with renal impairment [48], dabigatran is contraindicated in patients with CrCl <30 mL/min in the European Union. In 2010, the US Food and Drug Administration (FDA) approved dabigatran for the prevention of stroke and systemic embolism in patients with AF in two doses: 150 mg bid and, for patients with CrCl 15–30 mL/min, 75 mg bid [20]. In July 2011, the FDA added dabigatran to its list of drugs with a potential safety issue after reports of serious and fatal bleeding events in elderly patients with renal impairment [49]. For rivaroxaban, the approved dose in patients with AF and moderate (CrCl 30–49 mL/min) or severe (CrCl 15–29 mL/min) renal impairment is 15 mg od [4]. The approved dose of edoxaban in patients with moderate or severe renal impairment is 30 mg od [11]. However, data on NOACs in patients with estimated CrCl <30 mL/min are limited. For this reason, the latest ESC guidelines on AF recommend that none of the NOACs are used in this group of patients and that renal function is regularly monitored in all other patients [20]. Renal function should be assessed annually in patients within the normal CrCl range (≥80 mL/min) and in those with mild (CrCl 50–79 mL/min) impairment, and perhaps 2–3 times per year in patients with moderate (i.e., CrCl 30–49 mL/min) impairment [20].

### Patients with Indications for Antiplatelet Therapies

Antiplatelet therapy, including ASA and dual antiplatelet therapy (ASA plus clopidogrel/prasugrel/ticagrelor), is indicated in patients with a recent acute coronary syndrome (ACS). Approximately 15 % of patients with AF have concomitant ACS [50]. Standard antithrombotic therapy in the year after an ACS event currently comprises dual antiplatelet therapy (ASA plus a P2Y_12_ inhibitor), so patients with AF who have experienced an ACS event have indications for both anticoagulant and antiplatelet therapy [51–54]. Because the addition of antiplatelets to VKA therapy increases the risk of bleeding [55], safer options are needed for patients with concomitant AF and ACS. Although no clinical trial data are currently available to inform real-world practice with NOACs in this population, three studies, PIONEER AF-PCI, REDUAL-PCI, and AUGUSTUS, are currently underway. PIONEER AF-PCI is an exploratory, open-label, randomized, multicenter clinical study assessing the safety of two rivaroxaban strategies compared with VKA therapy in patients with AF who have undergone percutaneous coronary intervention with stent placement for ACS [56]. REDUAL-PCI is a randomized, open-label, blinded endpoint study currently recruiting patients with AF who have undergone percutaneous coronary intervention with stenting, to assess the efficacy and safety of two strategies of dabigatran therapy compared with VKA therapy (https://clinicaltrials.gov/ct2/show/NCT02164864). Lastly, AUGUSTUS, a randomized, open-label study with a 2 × 2 factorial design, is investigating the efficacy and safety of apixaban versus VKA and ASA therapy versus ASA placebo in patients with non-valvular AF who have undergone percutaneous coronary intervention with stent placement in the previous 14 days; all patients will also receive a P2Y_12_ inhibitor (https://clinicaltrials.gov/ct2/show/NCT02415400). Recommendations derived from expert consensus regarding the management of such patients can be found within current relevant European and US guidelines and the updated European Heart Rhythm Association practical guide on the use of NOACs in patients with non-valvular AF [57–59].

The relative benefit of dabigatran 110 mg bid versus warfarin for stroke/systemic embolism prevention was not affected by concomitant antiplatelet therapy [60]; however, a trend was observed for reduced efficacy with dabigatran 150 mg bid compared with warfarin (HR, 0.52 vs HR, 0.80; interaction *p* = 0.058). The relative efficacy of rivaroxaban and apixaban versus warfarin for prevention of stroke/systemic embolism was not affected by concomitant ASA therapy [61, 62]; likewise, concomitant antiplatelet therapy did not influence the relative efficacy of edoxaban versus warfarin [63]. As expected, in all four trials, concomitant treatment with a NOAC and antiplatelet therapy was associated with an increased incidence of bleeding events; however, there was no evidence of heterogeneity of safety outcomes between any of the NOACs versus warfarin [60–63].

### Differences in Phase III Trial Designs and Populations

Phase III trials provide compelling evidence that the NOACs have equal or superior efficacy and safety to warfarin in patients with AF, across a broad range of patient subtypes. Comparisons between the NOACs are complicated by important differences in study designs and patient populations in phase III trials.

ROCKET AF, ARISTOTLE, and ENGAGE AF used double-blind, double-dummy study designs, whereas patients in RE-LY received open-label warfarin. The open-label design of RE-LY may have been subject to bias, because patients randomized to the investigational drug were potentially more inclined to report minor signs or symptoms to the physicians, who may have interpreted them as adverse events of a novel agent. This might have been less likely to occur with the older and better established anticoagulant, warfarin.

Owing to prespecified study inclusion criteria, ROCKET AF enrolled a higher-risk patient population (mean CHADS_2_ score 3.5) compared with the other NOAC studies (ARISTOTLE and RE-LY: 2.1; ENGAGE AF: 2.8), in terms of co-morbidities [21–23, 25]. There were also notable differences in the methods of dose adjustment used in ENGAGE AF compared with other trials of NOACs. Patients assigned to edoxaban who were expected to experience increased drug exposure (because of one or more of the following: CrCl 30–50 mL/min, body weight ≤60 kg, or the concomitant use of verapamil, quinidine, or dronedarone) received a 50 % dose reduction at baseline, but dose adjustments were also permitted after randomization [25]. This contrasts with the RE-LY, ROCKET AF, and ARISTOTLE study designs, in which patients were allocated to the reduced dose only at baseline [21–23]. Therefore, direct comparisons between the NOAC trials should be avoided because the baseline characteristics of enrolled populations were very different.

Outcomes of the individual trials (Tables 4 and 5) provide important information to the clinicians, but indirect comparisons should be avoided because their validity is questionable and they should not be used to inform clinical decisions for individual patients.

## Practical Management

### Interactions with Rhythm-Controlling Drugs

Patients with AF frequently receive antiarrhythmic drugs such as amiodarone, verapamil, and dronedarone. These agents are inhibitors of P-glycoprotein and cytochrome P450 3A4, and because NOACs are substrates of one or both of these enzymes, interactions are expected. Caution is advised with the co-administration of antiarrhythmic agents, and some are contraindicated in patients prescribed a NOAC. There is a lack of data regarding the use of dronedarone in patients taking rivaroxaban, so co-administration should be avoided [4]. In ROCKET AF no significant interaction was observed between treatment effects in patients receiving amiodarone (8 % of enrolled patients) versus no antiarrhythmic drugs [64]. Dronedarone is contraindicated in patients taking dabigatran because it has been shown to increase plasma levels of dabigatran. Increases in dabigatran plasma concentration were also reported in patients co-administered amiodarone, quinidine, and verapamil [31]. Close clinical surveillance is recommended in patients receiving amiodarone or quinidine in combination with dabigatran, especially in patients with mild-to-moderate renal impairment. Patients receiving concomitant treatment with dabigatran and verapamil should receive the lower approved dose of dabigatran (110 mg bid) [31]. Amiodarone and verapamil have fewer significant interactions with apixaban, and in the ARISTOTLE trial there was no evidence of heterogeneity of outcomes between treatment groups in patients receiving amiodarone (11.4 % of enrolled patients) or not at randomization [8, 65]. In ENGAGE AF, the concomitant use of dronedarone, verapamil, or quinidine required the edoxaban dose to be halved for each dose group [25]. However, the edoxaban Summary of Product Characteristics only recommends an edoxaban dose reduction in patients receiving dronedarone, who should receive the lower approved dose (30 mg od); by contrast, no dose reduction is required for concomitant use of quinidine or verapamil [11]. In ENGAGE AF no dose reduction was required for patients receiving amiodarone (11.8 % of enrolled patients), and the relative efficacy and safety outcomes between patients receiving warfarin or the higher dose of edoxaban tested (60 mg od) were similar for patients with and without amiodarone use [66].

### Reversal and Anticoagulation Monitoring with Novel Oral Anticoagulants

The predictable pharmacokinetic/pharmacodynamic characteristics of the NOACs, including short half-lives and a wide therapeutic window, obviate the need for routine coagulation monitoring. However, there is a need to measure drug levels under certain circumstances; for example, possible overdose or drug accumulation, trauma, or suspected poor compliance [67]. The Hemoclot Thrombin Inhibitor assay, ecarin clotting time, and thrombin generation time assay are the most sensitive tests for measuring dabigatran anticoagulant effects; however, the activated partial thromboplastin time could also be used, because it has adequate sensitivity and is widely available [68]. Normal values of the activated partial thromboplastin time can exclude substantial overdosing of dabigatran. Anti-Factor Xa chromogenic assays such as Rotachrom® are recommended for the assessment of rivaroxaban, apixaban, and edoxaban [67, 69]. Such assays will use agent-specific calibrators and controls to measure plasma concentrations for the different Factor Xa inhibitors.

For reversal of anticoagulation, fresh–frozen plasma and prothrombin complex concentrates have been recommended as general strategies [70], but the former is poorly effective in the presence of substantial plasma concentrations of the active compounds. Other non-specific reversal agents include recombinant activated Factor VIIa (NovoSeven®) [71] and activated prothrombin complex concentrates (FEIBA®) [71]; however, these require testing in a clinical population and may be associated with a prothrombotic risk. Specific reversal agents for the NOACs are now in development. Idarucizumab, a monoclonal antibody that binds dabigatran and neutralizes its activity is at the most advanced stage of development; interim results from the REVERSE-AD trial have shown that it successfully reverses the anticoagulation effects of dabigatran in patients with serious bleeding or requiring emergency surgery [72]. On the basis of these findings it has recently received a positive recommendation from the Committee for Medicinal Products for Human Use (CHMP) committee at the European Medicines Agency (EMA) and is pending approval by the FDA [73]. Andexanet alfa (PRT064445) is a universal antidote for Factor Xa inhibitors that is currently in phase III development; results from phase III studies in elderly patients (ANNEXA-A and ANNEXA-R) show that it is capable of rapidly reversing the anticoagulant effects of apixaban and rivaroxaban [74, 75]. A third phase III study, recruiting patients receiving a Factor Xa inhibitor and experiencing acute major bleeding, is currently ongoing (https://clinicaltrials.gov/ct2/show/NCT02329327).

Although the very limited availability of specific reversal agents may be perceived as a current drawback of NOAC therapy, it should be remembered that reversing the effects of VKAs with vitamin K is slow and takes at least 24 h [76], which means that it is not clinically effective for serious bleeding events such as ICH. By contrast, the half-life of the NOACs rapidly removes their anticoagulation effect, which is likely to reduce the need for a specific reversal agent. Real-world evidence on the management of bleeding complications during rivaroxaban therapy from the Dresden NOAC Registry demonstrated that most cases of major bleeding events could be managed conservatively. The use of non-specific reversal agents was rare: out of the 66 major bleeding events (6.1 % of all bleeding events) reported in patients receiving rivaroxaban, prothrombin complex concentrate was used in six (9.1 %) of these patients [77].

## Conclusions

Because the incidence of AF is increasing [1] in a rapidly aging global population, AF-related stroke and its associated economic burden are expected to increase. Data from phase III trials indicate that the NOACs are at least as effective and safe as warfarin for stroke prevention in patients with non-valvular AF. NOACs also overcome some of the practical limitations seen with conventional VKA treatment and, therefore, may hold particular promise in challenging-to-treat patients, such as high-risk patients with multiple co-morbidities. Moreover, as demonstrated in AVERROES, the better efficacy and similar safety profile of apixaban versus ASA indicates that there are diminishing reasons for physicians to continue to prescribe ASA for stroke prevention in patients with non-valvular AF.

Encouragingly, emerging data from real-world clinical practice suggest that the increasing availability of the NOACs is correlated with a higher proportion of patients with non-valvular AF receiving OACs for stroke prevention [78], that there is improved treatment persistence with NOACs versus VKAs [79–81], and importantly, that real-world effectiveness and safety of the NOACs mirrors the findings of the phase III trials [82–85]. These data include recently published results from XANTUS, the first completed non-interventional phase IV study investigating the safety and efficacy of a NOAC in routine clinical practice, which showed that unselected patients with non-valvular AF treated with rivaroxaban experienced low rates of major bleeding (2.1 %/year) and stroke (0.7 %/year) over 1 year of follow-up [82]. Introduction of the NOACs is simplifying patient management, improving guideline adherence and increasing persistence. This is likely to increase the number of patients showing a favorable benefit–risk profile with NOACs, compared with warfarin, including a concomitant benefit regarding bleeding, especially ICH. Familiarization of cardiologists with the NOACs, and further information deriving from the large phase III trials and real-world studies, should help towards achieving this goal.

## Abbreviations

ARISTOTLE: Apixaban for reduction in stroke and other thromboembolic events in atrial fibrillation
AVERROES: Apixaban versus acetylsalicylic acid to prevent strokes
BAFTA: Birmingham atrial fibrillation treatment of the aged study
ENGAGE AF-TIMI 48: Effective anticoagulation with Factor Xa next generation in atrial fibrillation
RE-LY: Randomized evaluation of long-term anticoagulant therapy
ROCKET AF: Rivaroxaban once daily oral direct Factor Xa inhibition compared with vitamin K antagonism for prevention of stroke and embolism trial in atrial fibrillation
XANTUS: Xarelto® on prevention of stroke and non-central nervous system systemic embolism in patients with non-valvular atrial fibrillation

## References

[1] Camm AJ, Kirchhof P, Lip GYH (2010). Guidelines for the management of atrial fibrillation: the Task Force for the Management of Atrial Fibrillation of the European Society of Cardiology (ESC). Eur Heart J.

[2] Blech S, Ebner T, Ludwig-Schwellinger E, Stangier J, Roth W (2008). The metabolism and disposition of the oral direct thrombin inhibitor, dabigatran, in humans. Drug Metab Dispos.

[3] Stangier J, Stähle H, Rathgen K, Fuhr R (2008). Pharmacokinetics and pharmacodynamics of the direct oral thrombin inhibitor dabigatran in healthy elderly subjects. Clin Pharmacokinet.

[4] Bayer Pharma AG. Xarelto® (rivaroxaban) Summary of product characteristics. 2015. Available at: http://www.ema.europa.eu/docs/en_GB/document_library/EPAR_-_Product_Information/human/000944/WC500057108.pdf [accessed Sep 25, 2015].

[5] Kubitza D, Becka M, Wensing G, Voith B, Zuehlsdorf M (2005). Safety, pharmacodynamics, and pharmacokinetics of BAY 59–7939––an oral, direct Factor Xa inhibitor – after multiple dosing in healthy male subjects. Eur J Clin Pharmacol.

[6] Kubitza D, Becka M, Voith B, Zuehlsdorf M, Wensing G (2005). Safety, pharmacodynamics, and pharmacokinetics of single doses of BAY 59–7939, an oral, direct Factor Xa inhibitor. Clin Pharmacol Ther.

[7] Mueck W, Lensing AWA, Agnelli G, Décousus H, Prandoni P, Misselwitz F (2011). Rivaroxaban: population pharmacokinetic analyses in patients treated for acute deep-vein thrombosis and exposure simulations in patients with atrial fibrillation treated for stroke prevention. Clin Pharmacokinet.

[8] Bristol-Myers Squibb and Pfizer. Eliquis® (apixaban) Summary of product characteristics. 2015. Available at: http://www.ema.europa.eu/docs/en_GB/document_library/EPAR_-_Product_Information/human/002148/WC500107728.pdf [accessed Oct 16, 2015].

[9] Raghavan N, Frost CE, Yu Z (2009). Apixaban metabolism and pharmacokinetics after oral administration to humans. Drug Metab Dispos.

[10] Pinto DJ, Orwat MJ, Koch S (2007). Discovery of 1-(4-Methoxyphenyl)-7-oxo-6-(4-(2-oxopiperidin-1-yl)phenyl)-4,5,6,7-tetrahydro- 1H-pyrazolo[3,4-c]pyridine-3-carboxamide (Apixaban, BMS-562247), a highly potent, selective, efficacious, and orally bioavailable inhibitor of blood coagulation Factor Xa. J Med Chem.

[11] Daiichi Sankyo Europe GmbH. Lixiana® (edoxaban) Summary of product characteristics. 2015. Available at: http://www.ema.europa.eu/docs/en_GB/document_library/EPAR_-_Product_Information/human/002629/WC500189045.pdf [accessed Sep 25, 2015].

[12] Ogata K, Mendell-Harary J, Tachibana M (2010). Clinical safety, tolerability, pharmacokinetics, and pharmacodynamics of the novel Factor Xa inhibitor edoxaban in healthy volunteers. J Clin Pharmacol.

[13] Matsushima N, Lee F, Sato T, Weiss D, Mendell J (2013). Bioavailability and safety of the Factor Xa inhibitor edoxaban and the effects of quinidine in healthy subjects. Clin Pharmacol Drug Dev.

[14] Hart RG, Pearce LA, Aguilar MI (2007). Meta-analysis: antithrombotic therapy to prevent stroke in patients who have nonvalvular atrial fibrillation. Ann Intern Med.

[15] Bristol-Myers Squibb. Coumadin (warfarin sodium) Prescribing Information. 2011. Available at: http://packageinserts.bms.com/pi/pi_coumadin.pdf [accessed Jul 16, 2015].

[16] Kneeland PP, Fang MC (2010). Current issues in patient adherence and persistence: focus on anticoagulants for the treatment and prevention of thromboembolism. Patient Prefer Adhere.

[17] Kakkar AK, Mueller I, Bassand JP (2013). Risk profiles and antithrombotic treatment of patients newly diagnosed with atrial fibrillation at risk of stroke: perspectives from the international, observational, prospective GARFIELD Registry. PLoS One.

[18] You JJ, Singer DE, Howard PA (2012). Antithrombotic therapy for atrial fibrillation: antithrombotic therapy and prevention of thrombosis, 9th ed: American College of Chest Physicians evidence-based clinical practice guidelines. Chest.

[19] January CT, Wann LS, Alpert JS (2014). 2014 AHA/ACC/HRS Guideline for the management of patients with atrial fibrillation: a report of the American College of Cardiology/American Heart Association Task Force on Practice Guidelines and the Heart Rhythm Society. J Am Coll Cardiol.

[20] Camm AJ, Lip GYH, De Caterina R (2012). 2012 focused update of the ESC guidelines for the management of atrial fibrillation: an update of the 2010 ESC Guidelines for the management of atrial fibrillation. developed with the special contribution of the European Heart Rhythm Association. Eur Heart J.

[21] Connolly SJ, Ezekowitz MD, Yusuf S (2009). Dabigatran versus warfarin in patients with atrial fibrillation. N Engl J Med.

[22] Patel MR, Mahaffey KW, Garg J (2011). Rivaroxaban versus warfarin in nonvalvular atrial fibrillation. N Engl J Med.

[23] Granger CB, Alexander JH, McMurray JJ (2011). Apixaban versus warfarin in patients with atrial fibrillation. N Engl J Med.

[24] Connolly SJ, Eikelboom J, Joyner C (2011). Apixaban in patients with atrial fibrillation. N Engl J Med.

[25] Giugliano RP, Ruff CT, Braunwald E (2013). Edoxaban versus warfarin in patients with atrial fibrillation. N Engl J Med.

[26] Connolly SJ, Ezekowitz MD, Yusuf S, Reilly PA, Wallentin L (2010). Newly identified events in the RE-LY trial. N Engl J Med.

[27] Connolly SJ, Wallentin L, Yusuf S (2014). Additional events in the RE-LY trial. N Engl J Med.

[28] Lin HJ, Wolf PA, Kelly-Hayes M (1996). Stroke severity in atrial fibrillation. the Framingham Study. Stroke.

[29] Ruff CT, Giugliano RP, Braunwald E (2014). Comparison of the efficacy and safety of new oral anticoagulants with warfarin in patients with atrial fibrillation: a meta-analysis of randomised trials. Lancet.

[30] Nessel C, Mahaffey K, Piccini J (2012). Incidence and outcomes of gastrointestinal hemorrhage in patients with atrial fibrillation treated with rivaroxaban or warfarin: results from the ROCKET AF trial. Chest.

[31] Boehringer Ingelheim International GmbH. Pradaxa® (dabigatran etexilate) Summary of product characteristics. 2015. Available at: http://www.ema.europa.eu/docs/en_GB/document_library/EPAR_-_Product_Information/human/000829/WC500041059.pdf [accessed Oct 16, 2015].

[32] Bristol-Myers Squibb Company and Pfizer Inc. Eliquis® (apixaban) Prescribing information. 2015. Available at: http://packageinserts.bms.com/pi/pi_eliquis.pdf [accessed Oct 16, 2015].

[33] Fang MC, Go AS, Chang Y (2007). Death and disability from warfarin-associated intracranial and extracranial hemorrhages. Am J Med.

[34] Verma A, Cairns JA, Mitchell LB (2014). 2014 focused update of the Canadian Cardiovascular Society Guidelines for the management of atrial fibrillation. Can J Cardiol.

[35] Nieuwlaat R, Capucci A, Camm AJ (2005). Atrial fibrillation management: a prospective survey in ESC member countries: the Euro Heart Survey on Atrial Fibrillation. Eur Heart J.

[36] Go AS, Hylek EM, Borowsky LH, Phillips KA, Selby JV, Singer DE (1999). Warfarin use among ambulatory patients with nonvalvular atrial fibrillation: the AnTicoagulation and Risk Factors in Atrial Fibrillation (ATRIA) Study. Ann Intern Med.

[37] Boehringer Ingelheim Pharmaceuticals Inc. Pradaxa® (dabigatran etexilate) Prescribing Information. 2015. Available at: http://bidocs.boehringer-ingelheim.com/BIWebAccess/ViewServlet.ser?docBase=renetnt&folderPath=/Prescribing%20Information/PIs/Pradaxa/Pradaxa.pdf [accessed Oct 16, 2015].

[38] Janssen Pharmaceuticals Inc. Xarelto® (rivaroxaban) Prescribing Information. 2015. Available at: http://www.xareltohcp.com/sites/default/files/pdf/xarelto_0.pdf [accessed Oct 16, 2015].

[39] Daiichi Sankyo Inc. Savaysa® (edoxaban) Prescribing information. 2015. Available at: http://dsi.com/prescribing-information-portlet/getPIContent?productName=Savaysa&inline=true [accessed Oct 16, 2015].

[40] Singer DE, Chang Y, Fang MC (2009). The net clinical benefit of warfarin anticoagulation in atrial fibrillation. Ann Intern Med.

[41] Eikelboom JW, Wallentin L, Connolly SJ (2011). Risk of bleeding with 2 doses of dabigatran compared with warfarin in older and younger patients with atrial fibrillation: an analysis of the randomized evaluation of long-term anticoagulant therapy (RE-LY) trial. Circulation.

[42] Halperin JL, Wojdyla D, Piccini JP (2012). Efficacy and safety of rivaroxaban compared with warfarin among elderly patients with nonvalvular atrial fibrillation in the ROCKET-AF trial. Stroke.

[43] Halvorsen S, Atar D, Yang H (2014). Efficacy and safety of apixaban compared with warfarin according to age for stroke prevention in atrial fibrillation: observations from the ARISTOTLE trial. Eur Heart J.

[44] Kato ET, Giugliano RP, Ruff CT. Efficacy and safety of edoxaban for the managment of elderly patients with atrial fibrillation: Enage AF-TIMI 48. Circulation. 2014;130.

[45] Hijazi Z, Hohnloser SH, Oldgren J (2014). Efficacy and safety of dabigatran compared with warfarin in relation to baseline renal function in patients with atrial fibrillation: a RE-LY (Randomized Evaluation of Long-term Anticoagulation Therapy) trial analysis. Circulation.

[46] Fox KAA, Piccini JP, Wojdyla D (2011). Prevention of stroke and systemic embolism with rivaroxaban compared with warfarin in patients with non-valvular atrial fibrillation and moderate renal impairment. Eur Heart J.

[47] Hohnloser SH, Hijazi Z, Thomas L (2012). Efficacy of apixaban when compared with warfarin in relation to renal function in patients with atrial fibrillation: insights from the ARISTOTLE trial. Eur Heart J.

[48] Lehr T, Haertter S, Liesenfeld KH (2011). Dabigatran etexilate in atrial fibrillation patients with severe renal impairment: dose identification using pharmacokinetic modeling and simulation. J Clin Pharmacol.

[49] Food and Drug Administration. FDA Drug Safety Communication: Safety review of post-market reports of serious bleeding events with the anticoagulant Pradaxa (dabigatran etexilate mesylate). 2011. http://www.fda.gov/Drugs/DrugSafety/ucm282724.htm. Accessed Jul 16, 2015.

[50] Lau DH, Huynh LT, Chew DP, Astley CM, Soman A, Sanders P (2009). Prognostic impact of types of atrial fibrillation in acute coronary syndromes. Am J Cardiol.

[51] Roffi M, Patrono C, Collet JP. 2015 ESC Guidelines for the management of acute coronary syndromes in patients presenting without persistent ST-segment elevation: Task Force for the Management of Acute Coronary Syndromes in Patients Presenting without Persistent ST-Segment Elevation of the European Society of Cardiology (ESC). Eur Heart J. 2015.

[52] Steg PG, James SK, Atar D (2012). ESC guidelines for the management of acute myocardial infarction in patients presenting with ST-segment elevation: the Task Force on the Management of ST-Segment Elevation Acute Myocardial Infarction of the European Society of Cardiology (ESC). Eur Heart J.

[53] O’Gara PT, Kushner FG, Ascheim DD (2013). 2013 ACCF/AHA guideline for the management of ST-elevation myocardial infarction: a report of the American College of Cardiology Foundation/American Heart Association Task Force on Practice Guidelines. J Am Coll Cardiol.

[54] Amsterdam EA, Wenger NK, Brindis RG (2014). 2014 AHA/ACC guideline for the management of patients with non-ST-elevation acute coronary syndromes: a report of the American College of Cardiology/American Heart Association Task Force on Practice Guidelines. J Am Coll Cardiol.

[55] Hansen ML, Sørensen R, Clausen MT (2010). Risk of bleeding with single, dual, or triple therapy with warfarin, aspirin, and clopidogrel in patients with atrial fibrillation. Arch Intern Med.

[56] Gibson CM, Mehran R, Bode C (2015). An open-label, randomized, controlled, multicenter study exploring two treatment strategies of rivaroxaban and a dose-adjusted oral vitamin K antagonist treatment strategy in subjects with atrial fibrillation who undergo percutaneous coronary intervention (PIONEER AF-PCI). Am Heart J.

[57] Faxon DP, Eikelboom JW, Berger PB (2011). Consensus document: antithrombotic therapy in patients with atrial fibrillation undergoing coronary stenting. a North-American perspective. Thromb Haemost.

[58] Lip GYH, Windecker S, Huber K (2014). Management of antithrombotic therapy in atrial fibrillation patients presenting with acute coronary syndrome and/or undergoing percutaneous coronary or valve interventions: a joint consensus document of the European Society of Cardiology Working Group on Thrombosis, European Heart Rhythm Association (EHRA), European Association of Percutaneous Cardiovascular Interventions (EAPCI) and European Association of Acute Cardiac Care (ACCA) endorsed by the Heart Rhythm Society (HRS) and Asia-Pacific Heart Rhythm Society (APHRS). Eur Heart J.

[59] Heidbuchel H, Verhamme P, Alings M, et al. Updated European Heart Rhythm Association Practical Guide on the use of non-vitamin K antagonist anticoagulants in patients with non-valvular atrial fibrillation. Europace. 2015. 10.1093/europace/euv309 26324838

[60] Dans AL, Connolly SJ, Wallentin L (2013). Concomitant Use of Antiplatelet Therapy with Dabigatran or Warfarin in the Randomized Evaluation of Long-Term Anticoagulation Therapy (RE-LY) Trial. Circulation.

[61] Alexander JH, Lopes RD, Thomas L (2014). Apixaban vs. warfarin with concomitant aspirin in patients with atrial fibrillation: insights from the ARISTOTLE trial. Eur Heart J.

[62] Shah R, Hellkamp AS, Berkowitz SD. Rivaroxaban vs warfarin with concomitant aspirin use in patients with atrial fibrillation: findings from the ROCKET AF trial. Eur Heart J. 2015;36. 10.1016/j.ahj.2016.05.019 27595682

[63] Xu H, Ruff CT, Giugliano RP. Concomitant use of antiplately therapy with edoxaban or warfarin in patients with atrial fibrillation in the ENGAGE AF-TIMI 48 trial. Circulation. 2014;130.

[64] Steinberg BA, Hellkamp AS, Lokhnygina Y (2014). Use and outcomes of antiarrhythmic therapy in patients with atrial fibrillation receiving oral anticoagulation: results from the ROCKET AF trial. Heart Rhythm.

[65] Flaker G, Lopes RD, Hylek E (2014). Amiodarone, anticoagulation, and clinical events in patients with atrial fibrillation: insights from the ARISTOTLE trial. J Am Coll Cardiol.

[66] Steffel J, Giugliano RP, Braunwald E (2015). Edoxaban vs. warfarin in patients with atrial fibrillation on amiodarone: a subgroup analysis of the ENGAGE AF-TIMI 48 trial. Eur Heart J.

[67] Samama MM, Contant G, Spiro TE (2012). Evaluation of the anti-Factor Xa chromogenic assay for the measurement of rivaroxaban plasma concentrations using calibrators and controls. Thromb Haemost.

[68] Douxfils J, Mullier F, Robert S, Chatelain C, Chatelain B, Dogné JM (2012). Impact of dabigatran on a large panel of routine or specific coagulation assays. laboratory recommendations for monitoring of dabigatran etexilate. Thromb Haemost.

[69] Douxfils J, Mullier F, Loosen C, Chatelain C, Chatelain B, Dogné JM (2012). Assessment of the impact of rivaroxaban on coagulation assays: laboratory recommendations for the monitoring of rivaroxaban and review of the literature. Thromb Res.

[70] Makris M, Van Veen JJ, Tait C, Mumford A, Laffan M (2012). on behalf of the British Committee for Standards in Haematology. Guideline on the management of bleeding in patients on antithrombotic agents. Br J Haematol.

[71] Bauer KA (2012). Reversal of antithrombotic agents. Am J Hematol.

[72] Pollack CV, Reilly PA, Eikelboom J (2015). Idarucizumab for dabigatran reversal. N Engl J Med.

[73] European Medicines Agency. Committee for Medicinal Products for Human Use (CHMP) Summary of opinion (initial authorisation) Praxbind (idarucizumab). 2015. http://www.ema.europa.eu/docs/en_GB/document_library/Summary_of_opinion_-_Initial_authorisation/human/003986/WC500194147.pdf. Accessed Oct 14, 2015.

[74] Crowther M, Levy GG, Lu G. ANNEXA-A: a phase 3 randomized, double-blind, placebo-controlled trial, demonstrating reversal of apixaban-induced anticoagulation in older subjects by andexanet alfa (PRT064445), a universal antidote for Factor Xa (fXa) inhibitors. Circulation. 2014;130.

[75] Gold MA, Crowther M, Levy GG. ANNEXA-R: a phase 3 randomized, double-blind, placebo-controlled trial, demonstrating reversal of rivaroxaban-induced anticoagulation in older subjects by andexanet alfa (PRT064445), a universal antidote for Factor Xa (FXa) inhibitors. J Am Coll Cardiol. 2015;65.

[76] Hanley JP (2004). Warfarin reversal. J Clin Pathol.

[77] Beyer-Westendorf J, Förster K, Pannach S (2014). Rates, management, and outcome of rivaroxaban bleeding in daily care: results from the Dresden NOAC Registry. Blood.

[78] Kakkar AK. Global status of GARFIELD-AF. European Society of Cardiology congress. 2015.

[79] Zalesak M, Siu K, Francis K (2013). Higher persistence in newly diagnosed nonvalvular atrial fibrillation patients treated with dabigatran versus warfarin. Circ Cardiovasc Qual Outcomes.

[80] Nelson WW, Song X, Coleman CI (2014). Medication persistence and discontinuation of rivaroxaban versus warfarin among patients with non-valvular atrial fibrillation. Curr Med Res Opin.

[81] Martinez C, Katholing A, Wallenhorst C, Freedman SB. Therapy persistence in newly diagnosed non-valvular atrial fibrillation treated with warfarin or NOAC. a cohort study. Thromb Haemost. 2015;114. 10.1160/TH15-04-0350 26246112

[82] Camm AJ, Amarenco P, Haas S, et al. XANTUS: a real-world, prospective, observational study of patients treated with rivaroxaban for stroke prevention in atrial fibrillation. Eur Heart J. 2015. 10.1093/eurheartj/ehv466 PMC4823634 26330425

[83] Graham DJ, Reichman ME, Wernecke M (2015). Cardiovascular, bleeding, and mortality risks in elderly Medicare patients treated with dabigatran or warfarin for non-valvular atrial fibrillation. Circulation.

[84] Tamayo S, Frank PW, Patel M (2015). Characterizing major bleeding in patients with nonvalvular atrial fibrillation: a pharmacovigilance study of 27 467 patients taking rivaroxaban. Clin Cardiol.

[85] Lauffenburger JC, Farley JF, Gehi AK, Rhoney DH, Brookhart MA, Fang G et al. Effectiveness and safety of dabigatran and warfarin in real-world US patients with non-valvular atrial fibrillation: a retrospective cohort study. J Am Heart Assoc. 2015;4. 10.1161/JAHA.115.001798 PMC4579955 25862791

